# Automated Hematological Approach and Protein Electrophoretic Pattern in Tilapia (*Oreochromis niloticus*): An Innovative and Experimental Model for Aquaculture

**DOI:** 10.3390/ani14030392

**Published:** 2024-01-25

**Authors:** Francesco Fazio, Antonino Costa, Fabiano Capparucci, Gregorio Costa, Vincenzo Parrino, Francesca Arfuso

**Affiliations:** 1 Department of Veterinary Sciences, University of Messina, Via Palatucci, 98168 Messina, Italy; farfuso@unime.it; 2 Veterinary Practitioner, 98100 Messina, Italy; nino.costa88@libero.it; 3 Department of Chemical, Biological, Pharmaceutical and Environmental Sciences, University of Messina, 98166 Messina, Italy; fcapparucci@unime.it (F.C.); vparrino@unime.it (V.P.); 4 Department of Human Pathology in Adult and Developmental Age, University of Messina, 98125 Messina, Italy; gcosta@unime.it

**Keywords:** hematology, electrophoretic proteins, blood cells, farmed fish

## Abstract

**Simple Summary:**

Hematological analysis represents a valid tool for the control of pathologies and stress in fish, playing a crucial role in evaluating the health status of this animal species. Moreover, serum protein distribution and concentration can be affected by different physiological and pathological conditions. In view of such considerations, this study aimed to evaluate the usefulness of two innovative automated methods (automated blood count counter and flow cytometry) for hematological investigation and serum total proteins and their electrophoretic fractions in Nile Tilapia (*Oreochromis niloticus*). The findings obtained in the current study support the idea that the two hematological methods investigated could be useful in implementing the applicability of automated instrumental techniques in aquaculture.

**Abstract:**

This study aimed to assess the usefulness of two innovative automated methods (automated blood count counters and flow cytometry) for hematological investigation in Tilapia to make a contribution to the clinical diagnostics of this farmed species. Moreover, serum total proteins and their electrophoretic fractions (prealbumin, albumin, α-, β-, and γ-fraction), as health condition indicators, were assessed. The analysis of serum total proteins and electrophoretic fraction showed a normal and typical electrophoretic pattern of healthy fish (serum total proteins, 3.70 ± 0.62 g/dL; prealbumin, 0.44 ± 0.20 g/dL; albumin, 1.17 ± 0.66 g/dL; α-fraction, 1.49 ± 0.64 g/dL; β-fraction, 0.32 ± 0.16 g/dL; and γ-fraction, 0.29 ± 0.13 g/dL). The relationships between the values of red blood cells (RBCs), white blood cells (WBCs), and thrombocytes (TCs) obtained with the two automated methods were determined using Pearson correlation analysis. The results showed a significant positive correlation between automatic blood cell counting and flow cytometry analysis for RBCs (r = 0.97, *p* < 0.0001) and WBCs (r = 0.91, *p* < 0.0001), whereas no correlation was found for TCs (r = −0.11, *p* = 0.66). The preliminary results gathered in this study seem to highlight the usefulness of the new analytical techniques herein investigated in tilapia, suggesting their application in the hematological investigation of farmed fish species and their usefulness for monitoring the health and well-being of fish reared in aquaculture.

## 1. Introduction

Hematological profiles are commonly used in veterinary practice and scientific research, as well as to evaluate the health and welfare of fish in aquaculture. The elaboration of these profiles includes the evaluation of the blood cell count and is specifically based on the following parameters: The direct erythrocyte parameters, such as the number of red blood cells (RBCs), the hemoglobin concentration (Hb), the hematocrit value (Hct), and the indirect ones: the mean corpuscular volume (MCV), the mean corpuscular hemoglobin content (MCH), and the mean corpuscular hemoglobin concentration (MCHC). The numbers of white blood cells (WBCs) and thrombocytes (TCs) are also evaluated [1,2,3,4,5,6].

Differential blood cell count, including RBCs and WBCs, represents one of the suitable hematological indices of fish health as it could indicate the presence of an infectious disease [7] and could provide data regarding the pathogenesis and the defense mechanisms against disease adopted by fish [8,9]. Moreover, this approach has been used to assess fish responses to drug administration, parasite infestations, or environmental stress [10,11,12]. The blood with its constituents is a connective tissue that reflects several diseases; as a matter of fact, the abnormalities of morphology and number of RBCs, WBCs, and TCs are considered primary blood disorders. Indeed, it is well known that abnormalities in the function or structure of the blood cells may result in anemia, leukopenia, leukocytosis, neutropenia, thrombocytopenia, and other blood cell anomalies [13].

Hematological analysis in fish is inexpensive and can be evaluated with simple methods; among these methods, the most commonly used is the manual one since the morphology of blood cells does not allow automated determination as applied in mammals because all blood cells in fish, including red blood cells, are nucleated. Recently, attempts at automated hematological analyses have been carried out as an alternative to manual methods on fish blood [1,2,3,4,5]. The manual method for assessing hematological parameters in fish requires knowledge, skill, and experience in order to obtain reliable and reproducible results. Therefore, nowadays, there is an increasingly pressing need to improve and speed up the instrumental techniques in hematology applied to farmed fish species in order to guarantee valid support for the health monitoring of these species, the control of diseases, and the state of well-being, avoiding important economic losses in the aquaculture sector. Indeed, the application of automatic analysis methods for the evaluation of hematological parameters, as an alternative to manual methods, represents a valid diagnostic tool in aquaculture [5]. Evaluating the state of health in fish with innovative instruments capable of analyzing blood cell populations, such as automatic blood cell counting and flow cytometry, may represent the future of preventive fish medicine.

Flow cytometry (FCM) is a modern diagnostic method that allows the performance of blood cell counts and characterizes blood cell types in mixed populations with extraordinary speed and accuracy. Though this methodology is widely used in human hematological, pathological, and immunological research, its application in the study of fish blood is, to date, little used and not widespread. In veterinary medicine, the FCM method could represent an important diagnostic tool. The use of FCM for the detection and quantification of fish blood cells has been used in the European sea bass, *Dicentrarchus labrax* [6], the striped catfish, *Pangasius Hypothalamus* [14], the rainbow trout, *Oncorhynchus mykiss* [15], and the European eel, *Anguilla anguilla* [14]. A recent study [16] reports flow cytometric analyses applied to blood samples of two different teleost species (*Mugil cephalus* and *Carassius auratus*), highlighting a clear grouping of three main populations of blood cells, including blood cells (RBCs), white blood cells (WBCs), and thrombocytes (TCs). These cell types are closely related to the cell populations identified with the electronic cell counter [16]. Nile tilapia (*Oreochromis niloticus*) is among the most farmed fish species on the world market, with China, Indonesia, Egypt, and Brazil as the main producers. In Italy, the spread of this species in the aquaculture sector is severely limited by the difficulties in designing and building farms, which require specific characteristics in terms of the quality of fresh water and the size of the breeding tanks. *Oreochromis niloticus* is a euryhaline that can thrive from freshwater to full seawater. This fish species and its hybrids are good candidate species for cultivation in brackish habitats. The worldwide spread and growth of this species require greater knowledge of biology and techniques for monitoring the state of health through greater knowledge of the hematological profile and, at the same time, experimenting with the use of new and updated techniques and analytics to improve the accuracy of analyses. The number, morphology, and distribution of blood cells in fish are influenced by numerous endogenous and exogenous factors [17,18,19,20,21,22], which must be appropriately considered in farmed fish species. Modifications in the number and features of blood cells are useful indicators in the diagnosis of fish diseases [16], and thus, the use of automated and innovative analytical methods to evaluate them could provide significant enhancements to aquaculture practice and fisheries management. For this reason, the need to study the applicability of this technique to different fish species is well known.

The aim of this study was to assess the usefulness of an automatic hematology counter and a flow cytometer and to compare the two automated hematological techniques in Nile tilapia (*Oreochromis niloticus*) in order to improve the hematological knowledge of this teleost species’ helpfulness to evaluate the health status and well-being of fish as well as to implement automated instrumental techniques applicability in aquaculture.

## 2. Materials and Methods

### 2.1. Animals

For the study, a total of 18 Nile Tilapia (*Oreochromis niloticus*, Linnaeus 1758), coming from the “Ittica Caldoli” farm in the province of Foggia, Puglia (IT), were used. The enrolled fish showed an average weight of 240 ± 23 g and an average fork length of 23 ± 3 cm. The fish were transported in tanks with a volume of 80 L (70 × 34 × 40 cm), equipped with a thermostat and oxygenator, in order to minimize transport stress and mortality. Upon arrival in the animal facility, fish were acclimatized for 25 days before the experimental test in tanks of approximately 400 L (140 × 50 × 63 cm), appropriately disinfected and equipped with a biological filter, and also added approximately 50 mL of “Bacter Plus” (Aqua Design Amano Co., Ltd., Niigata, Japan). The tank has been enriched with small hiding places, as well as an oxygenator, to help the fish acclimatize and reduce stress to a minimum. The water parameters evaluated during the study were as follows: temperature 20 °C, salinity 0.3 ‰, conducibility 620 μS/cm, pH 7.56, and O_2_ saturation 97%. The animals involved in the study were housed at the Aquatech supplier plant at the University of Messina, the Department of ChiBioFarAm, company code IT048ME157, dated 15/2/23. All fish were fed feed that had the following characteristics: 50% crude protein; 16% crude lipids; 3.5% crude fiber; 6% ash; 0.2% sodium; and 0.3% calcium. No mortality of fish occurred during the acclimation period. Moreover, all fish enrolled in the study were screened, and results showed that all were free from external parasites and without lesions.

### 2.2. Blood Sampling

Blood samples were taken during routine health monitoring of the fish under the supervision of the animal welfare manager and the veterinarian.

Before taking blood samples, the fish were anesthetized with 2-phenoxyethanol at a concentration of 0.4 g/L of water. The blood samples were collected from the caudal vein (always in the morning at the same time between 08.00 and 09.00) via venipuncture using a sterile 2.5 mL plastic syringe and transferred into test tubes (Miniplast 0.6 mL; LP Italiana Spa, Milan, Italy) containing EDTA (ethylenediaminetetraacetic acid) (ratio 1.26 mg/0.6 mL) as an anticoagulant agent for the determination of the hematological profile, and in tubes without anticoagulant for the evaluation of total serum proteins and their fractions.

Immediately after blood sampling, the biometric indices (weight, fork length, and total length) of each fish were measured; for this purpose, a scale (Kern 440-49 N, Balingen, Germany) and an ichthyometer (Scubla SNC, 600 mm, Remanzacco, Italy) were used, respectively. From the external examination, all subjects were declared clinically healthy due to the absence of external lesions and parasites.

Blood samples collected into EDTA tubes were analyzed within 5 h of collection through an automatic hematology counter and a flow cytometer for the evaluation of red blood cells (RBCs), white blood cells (WBCs), and thrombocytes (TCs). All analyses were performed in triplicate with the same operator and with the same instrument. No significant differences were found between the three measurements, whereas blood collected into tubes without an anticoagulant agent, following standing at room temperature for 20 min, was centrifuged at room temperature at 1300× *g* for 10 min, and the obtained serum was stored at −20 °C until analyzed.

### 2.3. Automated Blood Count Test

For the determination of hematological parameters (white blood cells, WBCs; red blood cells, RBCs; hematocrit, Hct; hemoglobin concentration, Hb; mean corpuscular volume, MCV; mean corpuscular hemoglobin content, MCH; mean corpuscular hemoglobin concentration, MCHC; and thrombocyte cells, TCs), an electronic blood cell counter (HecoVet, SEAC, Florence, Italy) was used. The instrument was already used in the veterinary field for mammals and appropriately modified in the software in order to carry out hematological analyses on fish species with reliable results as described in previous studies [5,23].

### 2.4. Flow Cytometer Test

Flow cytometric analysis was performed using a multispectral flow cytometer combining standard microscopy with flow cytometry (ImageStreamX) (Amnis) (Fremont, CA, USA) according to previous studies on other fish species [24,25].

This instrument allows the simultaneous acquisition of six images (including bright field, scatter, and multiple fluorescent images) of each cell (up to 100 cells/s). For the present study, the integrated INSPIRE software (IDEAS Application Version 6.2.187.0), running on ImageStreamX Mark II, was applied. Before injecting the samples into the flow cell, they were kept on ice. Then, cells were allowed to form a single nucleus stream for acquisition. Images were analyzed using IDEAS image analysis software (Amnis) (Version 200.1.280.0).

### 2.5. Serum Total Proteins and Their Fractions Analysis

Serum total protein concentration was assessed by means of an automated UV spectrophotometer (Slim, SEAC, Florence, Italy) using the Biuret method with a commercially available kit (Biosystems S.A., Barcelona, Spain). An automated system (Selvet24, Seleo Engineering, Naples, Italy) was used, according to the procedures described by the manufacturer and as previously described [26], to assess protein fractions. The major protein fractions were divided into prealbumin, albumin, α-, β-, and γ-globulins, from the cathode to the anode, according to the recommendation by the manufacturer and in agreement with previous findings gathered in fish species [26]. Relative protein concentrations within each fraction were determined as the optical absorbance percentage, and then the absolute concentration (g/dL) was calculated using the total protein concentration.

### 2.6. Statistical Analysis

Data were screened for normality by means of the Kolmogorov–Smirnov test, and they were normally distributed (*p* > 0.05). The relationships between blood cell values obtained using the two analytical methods were investigated using Pearson’s correlation analysis. A linear regression model (y = a + bx) was applied to analyze the correlation degree. *p* values < 0.05 were considered statistically significant. The statistical analysis was performed using the software Prism v. 9.00 (Graphpad Software Ltd., La Jolla, CA, USA, 2020).

## 3. Results

The obtained data are presented as means ± standard deviation (SD).

### 3.1. Serum Protein

**Figure 1:**
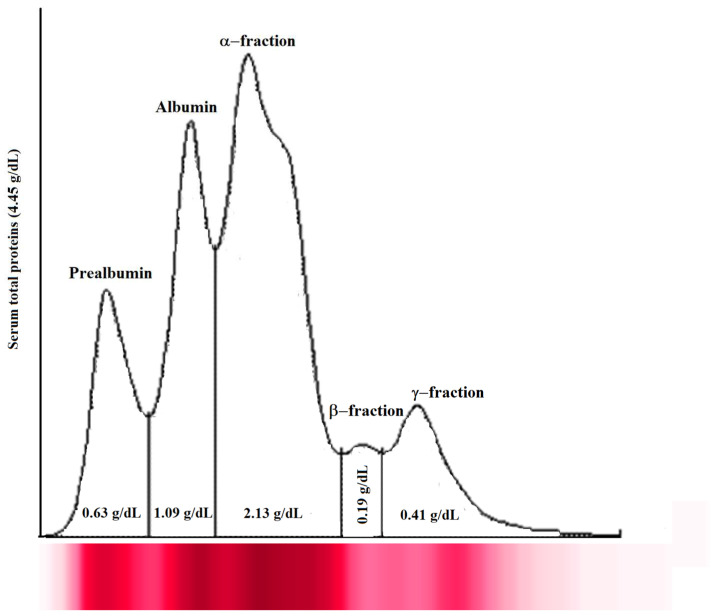
Representative serum protein electrophoretogram observed in Tilapia investigated in the study. The major protein fractions were divided into prealbumin, albumin, α-fraction, β-fraction, and γ-fraction, from the cathode to the anode.

The analysis of serum total proteins and electrophoretic fraction showed a normal and typical electrophoretic pattern of healthy fish (serum total proteins, 3.70 ± 0.62 g/dL; prealbumin, 0.44 ± 0.20 g/dL; albumin, 1.17 ± 0.66 g/dL; α-fraction, 1.49 ± 0.64 g/dL; β-fraction, 0.32 ± 0.16 g/dL; and γ-fraction, 0.29 ± 0.13 g/dL). A representative serum protein electrophoretogram observed in Tilapia enrolled in this study is shown in Figure 1.

### 3.2. Hematological Analysis

**Figure 2:**
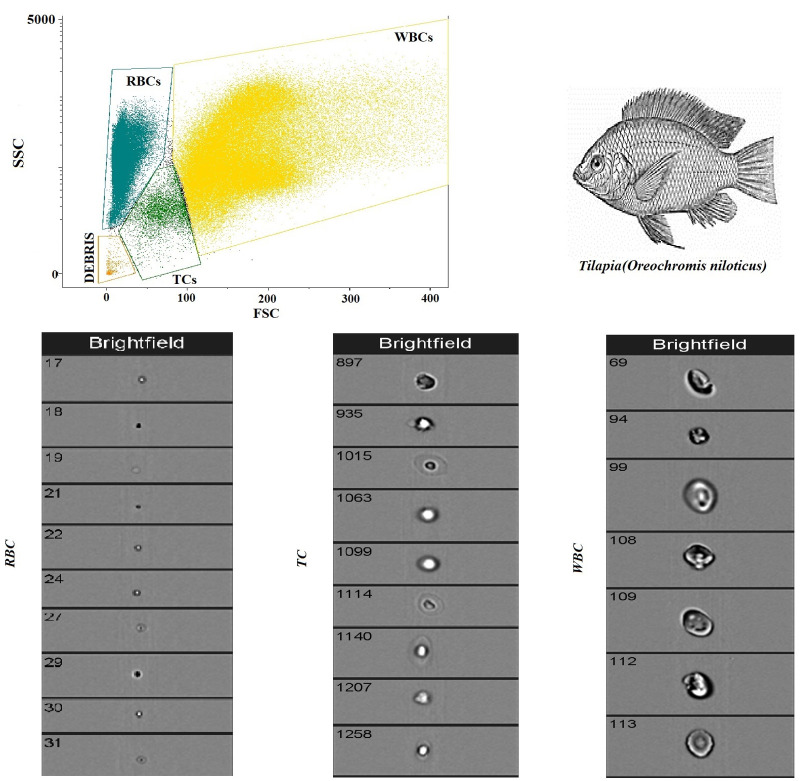
Flow cytometry analysis of blood cells with cell population counting in Tilapia.

**Figure 3:**
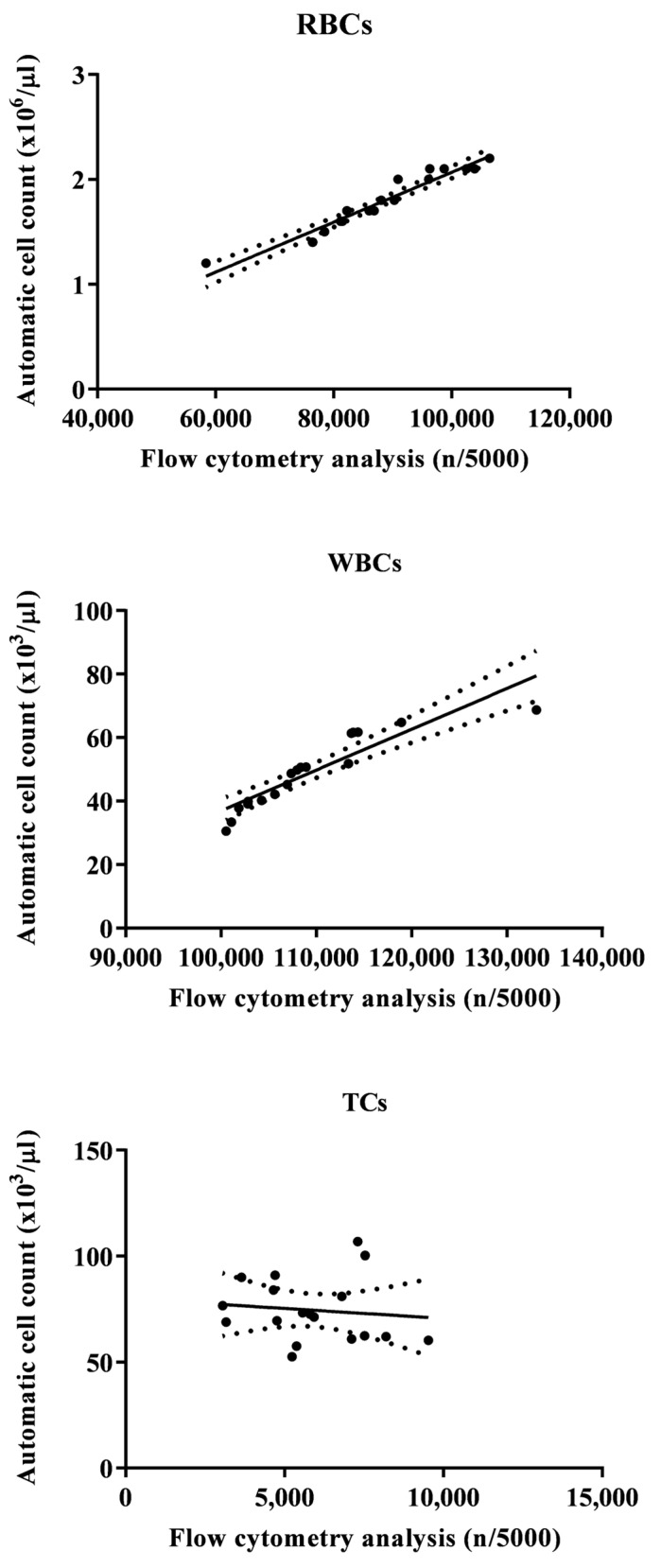
Flow statistically significant linear regression between the values of red blood cells (RBCs), white blood cells (WBCs), and thrombocytes (TCs) evaluated in Tilapia (*Oreochromis niloticus*) using the automatic blood cell count and the flow cytometry analysis.

Figure 2 shows a flow cytometry analysis of blood cells with cell population counting in Tilapia. In particular, flow cytometric analysis shows that, out of a total of 5000 cells counted, the largest cell population is represented by RBCs, followed by WBCs and TCs. Table 1 shows the complete hematological profile obtained using an electronic blood cell counter. Table 2 shows the mean values ± SD of the hematological cells (RBCs, WBCs, and TCs) in Tilapia, together with Pearson’s correlation results, studied in Tilapia using the automatic blood cell counter and the flow cytometry analysis. Specifically, statistical analysis of the data showed a positive correlation between the two analytical methods only for WBCs and RBCs (*p* < 0.0001), whereas no correlation was found for TCs. These results were confirmed using the linear regression model (Figure 3).

## 4. Discussion

Hematological investigations in farmed fish, like all animals used in the production sector, represent the basic tool for the assessment of the health status and well-being of the animals [27]. However, a deep knowledge of environmental factors as well as the physical and chemical properties of water is crucial to best interpreting the variations in hematological parameters and evaluating fish health status [28]. In this regard, temperature, salinity, and pH of water, the photoperiod, and the farming system affect the hematological parameters in captive fish. Moreover, it has been demonstrated that the availability of food, as well as its type, may even influence erythropoiesis in fish [29,30]; therefore, this aspect must be considered during a hematological assessment. In the last few years, the development of new methods for the differentiation and quantification of blood cells in fish species has acquired great interest; however, the fish veterinary sector is still distant from the methods and instruments that are widely used for the evaluation of hematological parameters in human and mammals’ species. Nowadays, it is necessary to identify a reference range for the farmed fish species and an automated evaluation system that can speed up hematological investigations with reliable results.

According to the results gathered in the current study, the Tilapia specimens herein investigated showed a serum protein electrophoretic pattern similar to that found in other fish species, showing five fractions, including prealbumin, albumin, α-fraction, β-fraction, and γ-fraction [26]. The protein electrophoretic pictures obtained from each fish appear to be consistent with the state of health of animals [26]. As a matter of fact, the assessment of the serum protein electrophoretic pattern allows us to investigate the inflammatory and immune responses. Following electrophoresis, serum proteins can be separated into several fractions with different physiological and metabolic properties. The identification and quantification of protein fractions enable the identification of animals with altered serum protein patterns reflecting their response to homeostasis disruption or disease.

In the current study, the use of flow cytometry and automated methods as electronic blood cell counters was tested for the hematology of Tilapia. The flow cytometric method allows the fast detection of different blood cell types within the whole peripheral blood. Specifically, this technique allows for the discrimination of different cell populations based on forward light scattering and lateral scattering, providing a rapid method for differential cell counting. Therefore, this method offers significant advantages compared to traditional hematological techniques, thanks to the speed and accuracy of the analysis. However, no definitive flow cytometry data exist for blood cells in fish; therefore, expanding studies on this field, including many fish species, is demanded. The results of this study showed a well-defined pattern of blood cells through flow cytometry analysis in Tilapia. Flow cytometric analysis shows that, out of a total of 5000 cells counted, the largest cell population is represented by RBCs (n = 87,820.9), followed by WBCs (n = 10,922.5) and TCs (n = 5883.3). Previous studies carried out in fish showed the usefulness of flow cytometry in the quantitative analysis and differentiation of peripheral blood leukocytes in the common carp [31,32,33,34], of the different cell types in sea bass [7], in salmonids [15], and in tilapia [35]. Some authors [36] have highlighted that flow cytometric analysis of blood cells can be considered a biomarker to evaluate fish toxicity. A recent study [8] reports on flow cytometric analyses applied to blood samples of two different teleosts (*Mugil cephalus* and *Carassius auratus*), highlighting a clear grouping of three main populations of blood cells, such as RBCs, WBCs, and TCs, closely related to the cell populations identified with the electronic cell counter. The usefulness of flow cytometry has been suggested to estimate cellular activity and cellular immune response; in this regard, it has been proven that the measurement performed using this method is on the single cell and not on the whole cell population, and, thus, it is possible to associate the cell function or reaction with one sub-population [37].

The automated evaluation of hematological parameters has been studied by many authors in different species; some authors [5] compared the results obtained using both the manual method and the automated system (electronic blood cell counter type HeCo Vet C, SEAC, Florence, Italy) with the indications relating to a special lysant for fish and software integrated into the blood cell counter for reading *Sparus aurata* blood samples. The studies showed no significant differences in RBCs, WBCs, and TCs between samples analyzed using the automated method and samples analyzed using the traditional method. Filiciotto et al. [38] demonstrated the high reliability of the same automatic method for the blood of sea bass. Similarly, Faggio et al. [39] showed no significant differences between the results from blood samples analyzed manually and automatically (by means of HeCo Vet C) of *M. cephalus.* Yilmaz and Ergün [40] showed no statistically significant differences in red blood cell levels determined in the blood of *Oncorhynchus mykiss* using the manual method and automatic blood count instrument (Mindray BC 3000 plus, Shenzhen, China). Automatic blood analyzers (HeCo Vet C and Mindray BC 2800 Vet) were also used for the analysis of the blood parameters of *O. mykiss*. It appears that, despite the fact that automatic hematological analysis methods seem to be appropriate for fish blood analyses, they should be used with caution. Specifically, the use of automated hematological methods should be validated through traditional manual analyses carried out by an expert in the cellular morphology of fish blood.

The results gathered in the current study showed a positive relationship between automatic blood cell counting and flow cytometry analysis for WBCs and RBCs in Tilapia, while no correlation was found for TCs. These data confirm what has already been found in other species, such as the Striped Bass (*Morone Saxatilis*) [5]. Noteworthy, the two automated systems (blood cell counter and flow cytometer) gave comforting results for RBCs and WBCs in both tilapia and Striped Bass, showing a close association between the values of these blood cell types obtained using the two methods. The results obtained in the current study support the effectiveness of electronic blood cell counters and flow cytometry in the hematological investigation, or at least for RBCs and WBCs, in fish species; furthermore, the findings herein obtained suggest the usefulness of these automated methods for health control and for monitoring the state of welfare and well-being in farmed fish species. It is necessary to understand the reason for the missing correlation between the values of TCs obtained using these two automated systems in tilapia as well as in Striped Bass. Likely, the reason for the lack of correlation for TCs is linked to the morphological variability of this blood cell population. Indeed, fish TCs are small cells with an oblong, ovoid, or spindle shape (these morphological shapes can be observed in the same blood sample), clear cytoplasm, and a highly condensed basophilic nucleus. Furthermore, in different fish species, TCs show wide variability in shape and can be round, oval, point-shaped, spindle-shaped, or elongated [41,42,43].

## 5. Conclusions

The continuously expanding aquaculture sector requires systems to control and monitor the health status of farmed fish. The results obtained in this study, although preliminary, validate the use of new analytical techniques that can be applied in the hematology of farmed fish species, which could be useful for health control and for monitoring the state of welfare and well-being of fish. Automated systems such as electronic blood cell counters and flow cytometry may represent valid diagnostic aids for the evaluation of hematological parameters in fish due to the speed and reliability of these methods. The usefulness and accuracy of these techniques in other farmed fish species are worthy of investigation, in view of the fact that their use could make a notable contribution to the investigation of hematological parameters, which are scant in the literature for farmed fish species.

## Figures and Tables

**Table 1:** Mean values ± standard deviation (±SD) of hemoglobin concentration (Hb), hematocrit (Hct), mean corpuscular volume (MCV), mean corpuscular hemoglobin content (MCH), and mean corpuscular hemoglobin concentration (MCHC) evaluated in Tilapia (*Oreochromis niloticus*) using the automatic blood cell counter.

Hematological Parameters	Mean ± DS
**Hb (g/dL)**	9.19 ± 1.76
**Hct (%)**	25.76 ± 4.58
**MCV (fL)**	145.18 ± 7.50
**MCH (Pg)**	5.80 ± 4.37
**MCHC (%)**	35.66 ± 2.13

**Table 2:** Mean values ± standard deviation (±SD) together with Pearson’s correlation results of red blood cells (RBCs), white blood cells (WBCs), and thrombocytes (TCs) evaluated in Tilapia (*Oreochromis niloticus*) using the automatic blood cell counter and the flow cytometry analysis.

Parameters	Automated Blood Cell Counter	Flow Cytometer (n/5000 Total Cells)	Pearson’s Correlation Results
**RBCs**	1.77 ± 0.31 (×10^6^/μL)	87,820.9 ± 11,950.79	r = 0.97, *p* < 0.0001
**WBCs**	48.78 ± 11.26 (×10^3^/μL)	10,922.5 ± 7952.22	r = 0.91, *p* < 0.0001
**TCs**	74.55 ± 15.13 (×10^3^/μL)	5883.28 ± 1778.90	r = −0.11, *p* = 0.66

## Data Availability

The data presented in this study are available on request from the corresponding author. The data are not publicly available due to privacy reason.

## References

[1] Tavares-Dias M., Ruas de Moraes F. (2006). Hematological parameters for the *Brycon orbignyanus* Valenciennes, 1850 (Osteichthyes, Characidae) intensively bred. Hidrobiology.

[2] Fazio F., Faggio C., Marafoti S., Torre A., Sanflippo M., Piccione G. (2013). Effect of water quality on hematological and biochemical parameters of *Gobius niger* caught in Faro lake (Sicily). Iran. J. Fish. Sci..

[3] Różyński M., Demska-Zakęs K., Sikora A., Zakęs Z. (2018). Impact of inducing general anesthesia with Propiscin (etomidate) on the physiology and health of European perch (*Perca fluviatilis* L.). Fish Physiol. Biochem..

[4] Fazio F. (2019). Fish hematology analysis as an important tool of aquaculture: A review. Aquaculture.

[5] Fazio F., Saoca C., Costa G., Zumbo A., Piccione G., Parrino V. (2019). Flow cytometry and automatic blood cell analysis in striped bass *Morone saxatilis* (Walbaum, 1792): A new hematological approach. Aquaculture.

[6] Esteban M.A., Muñoz J., Meseguer J. (2000). Blood cells of sea bass (*Dicentrarchus labrax* L.). Flow cytometric and microscopic studies. Anat. Rec..

[7] Grant K.R. (2015). Fish hematology and associated disorders. Vet. Clin. Exot. Anim..

[8] Fijan N. (2002). Morphogenesis of blood cell lineages in channel catfish. J. Fish Biol..

[9] Fijan N. (2002). Composition of main haematopoietic compartments in normal and bled channel catfish. J. Fish Biol..

[10] Pavlidis M., Futter W.C., Katharios P., Divanach P. (2007). Blood cell profile of six Mediterranean mariculture fish species. J. Appl. Ichthyol..

[11] Zexia G., Weimin W., Yi Y., Abbas K., Dapeng L., Guiwei Z., Diana J.S. (2007). Morphological studies of peripheral blood cells of the Chinese sturgeon, *Acipenser sinensis*. Fish Physiol. Biochem..

[12] El-Naggar A.M., El-Tantawy S.A., Mashaly M.I., Kanni A. (2017). Reproductive behaviour, hematological profile and monogenean microfauna of the Nest-breeding, Nile green Tilapia (*Tilapia zilli*) Gervais, 1848. J. Environ. Sci. Toxicol. Food Technol..

[13] Clauss T.M., Dove A.D.M., Arnold J.E. (2008). Hematologic disorder of fish. Vet. Clin. North Am. Exot. Anim. Pract..

[14] Pierrard M.-A., Roland K., Kestemont P., Dieu M., Raes M., Silvestre F. (2012). Fish peripheral blood mononuclear cells preparation for future monitoring applications. Anal. Biochem..

[15] Morgan J.A.W., Pottinger T.G., Rippon P. (1993). Evaluation of flow cytometry as a method for quantification of circulating blood cell populations in salmonid fish. J. Fish Biol..

[16] Parrino V., Cappello T., Costa G., Cannavà C., Sanfilippo M., Fazio F., Fasulo S. (2018). Comparative study of haematology of two teleost fish (*Mugil cephalus* and *Carassius auratus*) from different environments and feeding habits. Eur. Zool. J..

[17] May-Tec A.L., Ek-Huchim J.P., Rodríguez-González A., Mendoza-Franco E.F. (2023). Differential blood cells associated with parasitism in the wild puffer fish *Lagocephalus laevigatus* (Tetraodontiformes) of the Campeche Coast, southern Mexico. Parasitol. Res..

[18] De Oliveira-Lima J., Dias da Cunha R.L., de Brito-Gitirana L. (2022). Effect of benzophenone-3 on the blood cells of zebrafish (*Danio rerio*). J. Environ. Sci. Health B..

[19] Pierrard M.A., Kestemont P., Phuong N.T., Tran M.P., Delaive E., Thezenas M.L., Dieu M., Raes M., Silvestre F. (2012). Proteomic analysis of blood cells in fish exposed to chemotherapeutics: Evidence for long term effects. J. Proteom..

[20] Bhardwaj A.K., Chandra R.K., Pati A.K., Tripathi M.K. (2022). Seasonal immune rhythm of leukocytes in the freshwater snakehead fish, *Channa punctatus*. J. Comp. Physiol. B.

[21] Ahmed I., Reshi Q.M., Fazio F. (2020). The influence of the endogenous and exogenous factors on hematological parameters in different fish species: A review. Aquac. Int..

[22] Alvarez-Pellitero P., Pinto R.M. (1987). Some blood parameters in sea bass, *Dicentrarchus labrax*, infected by bacteria, virus and parasites. J. Fish Biol..

[23] Anyanwu P.E., Gabriel U.U., Anyanwu A.O. (2007). Effect of salinity changes on haematological parameters of *Sarotherodon melanotheron* from Buguma Creek, Niger Delta. J. Anim. Vet. Adv..

[24] Yavuzcan Y.H., Bekcam S., Karasu Benli A.C., Akan M. (2005). Some blood parameters in the eel (*Anguilla anguilla*) spontaneously infected with *Aeromonas hydrophila*. Aquac. Res..

[25] Fazio F., Filiciotto F., Marafioti S., Di Stefano V., Assenza A., Placenti F., Buscaino G., Piccione G., Mazzola S. (2012). Automatic analysis to assess haematological parameters in farmed gilthead sea bream (*Sparus aurata* Linnaeus, 1758). Mar. Freshw. Behav. Physiol..

[26] Faggio C., Piccione G., Marafioti S., Arfuso F., Fortino G., Fazio F. (2014). Metabolic response to monthly variations of *Sparus aurata* reared in Mediterranean off-shore tanks. Turk. J. Fish. Aquat. Sci..

[27] Mauri I., Romeo A., Acerete L., Mackenzie S., Roher N., Callol A., Cano I., Alvarez M.C., Tort L. (2011). Changes in complement responses in gilthead seabream (*Sparus aurata*) and European seabass (*Dicentrarchus labrax*) under crowding stress, plus viral and bacterial challenges. Fish Shellfish Immunol..

[28] Bosisio F., Fernandes Oliveira Rezende K., Barbieri E. (2017). Alterations in the hematological parameters of juvenile Nile Tilapia (*Oreochromis niloticus*) submitted to different salinities. Pan-Am. J. Aquat. Sci..

[29] Řehulka J., Adamec V. (2004). Red blood cell indices for rainbow trout (*Oncorhynchus mykiss* Walbaum) reared in cage and raceway culture. Acta Vet. Brno.

[30] Burgos-Aceves M.A., Lionetti L., Faggio C. (2019). Multidisciplinary haematology as prognostic device in environmental and xenobiotic stress-induced response in fish. Sci. Total Environ.

[31] Fazio F., Costa G., Piccione G., Giannetto C., Parrino V., Arfuso F. (2022). Innovative approach for haematological analysis in *Gobius niger* (Linnaeus, 1758) and *Mugil cephalus* (Linnaeus, 1758): Useful model in fish preventive medicine. Aquac. Res..

[32] Inoue T., Moritomo T., Tamura Y., Mamiya S., Fujino H., Nakanishi T. (2002). A new method for fish leucocyte counting and partial differentiation by flow cytometry. Fish Shellfish Immunol..

[33] Korytář T., Dang Thi H., Takizawa F., Köllner B.A. (2013). A multicolour flow cytometry identifying defined leukocyte subsets of rainbow trout (*Oncorhynchus mykiss*). Fish Shellfish Immunol..

[34] Stosik M., Deptula W., Wiktorowicz K., Travnicek M., Baldy-Chudzik K. (2001). Qualitative and quantitative cytometric analysis of peripheral blood leukocytes in carps (*Cyprinus carpio*). Vet. Med..

[35] Gomes J.M.M., Charlie-Silva I., Santos A.K., Resende R.R., Gomes J.A.S., de Carvalho A.T., Corrêa Junior J.D. (2021). Flow cytometry in the analysis of hematological parameters of tilapias: Applications in environmental aquatic toxicology. Environ. Sci. Pollut. Res. Int..

[36] Chilmonczyk S., Monge D. (1999). Flow cytometry as a tool for assessment of the fish cellular immune response to pathogens. Fish Shellfish Immunol..

[37] Maitra B., Sen S., Homechaudhuri S. (2014). Flow cytometric analysis of fish leukocytes as a model for toxicity produced by azadirachtin-based bioagrocontaminant. Toxicol. Environ. Chem..

[38] Filiciotto F., Fazio F., Marafioti S., Buscaino G., Maccarrone V., Faggio C. (2012). Assessment of haematological parameters range values using an automatic method in European sea bass (*Dicentrarchus labrax* L.). Nat. Rerum.

[39] Faggio C., Casella S., Arfuso F., Marafioti S., Piccione G., Fazio F. (2013). Effect of storage time on haematological parameters in mullet, *Mugil cephalus*. Cell Biochem. Funct..

[40] Yilmaz S., Ergün S. (2018). Trans-cinnamic acid application for rainbow trout (*Oncorhynchus mykiss*): I. Effects on haematological, serum biochemical, non-specific immune and head kidney gene expression responses. Fish Shellfish Immunol..

[41] Lopez-Ruiz A., Esteban M.A., Meseguer J. (1992). Blood cells of the gilthead seabream (*Sparus aurata* L.): Light and electron microscopic studies. Anat. Rec..

[42] Sardar M., Mahna K., Alam M., Mamnur Rashid M. (2000). Cell types in the peripheral blood of waking catfish *Clarias batrachus* (Lin.). Bangladesh Fish. Res..

[43] Tavares-Dias M., Ono E.A., Pilarski F., Moraes F.R. (2007). Can thrombocytes participate in the removal of cellular debris in the blood circulation of teleost fish? A cytochemical study and ultrastructural analysis. J. Appl. Ichthyol..

